# The Shifting Paradigm of Prognostic Factors of Colorectal Liver Metastases: From Tumor-Centered to Host Immune-Centered Factors

**DOI:** 10.3389/fonc.2018.00181

**Published:** 2018-05-28

**Authors:** Matteo Donadon, Ana Lleo, Luca Di Tommaso, Cristiana Soldani, Barbara Franceschini, Massimo Roncalli, Guido Torzilli

**Affiliations:** ^1^Department of Hepatobiliary and General Surgery, Humanitas Clinical and Research Center, Rozzano, Italy; ^2^Department of Biomedical Science, Humanitas University, Rozzano, Italy; ^3^Department of Internal Medicine, Humanitas Clinical and Research Center, Rozzano, Italy; ^4^Department of Pathology, Humanitas Clinical and Research Center, Rozzano, Italy

**Keywords:** host immune system, colorectal liver metastases, colorectal cancer liver metastases, colorectal cancer, immune response, immunotherapy

## Abstract

The determinants of prognosis in patients with colorectal liver metastases (CLM) have been traditionally searched among the tumoral factors, either of the primary colorectal tumor or of the CLM. While many different scoring systems have been developed based on those clinic-pathological factors with disparate results, there has been the introduction of genetic biological markers that added a theranostic perspective. More recently, other important elements, such as those factors related to the host immune system, have been proposed as determinants of prognosis of CLM patients. In the present work, we review the current prognostic factors of CLM patients as well as the burgeoning shifting paradigm of prognostication that relies on the host immune system.

## Introduction

Colorectal cancer is the third most common cancer as well as the second cause of cancer-related deaths worldwide (1). Being the liver the main filter of the venous drainage of the bowel, most of the patients with colorectal cancer develop colorectal liver metastases (CLM). Hepatic resection for CLM combined with systemic chemo-immunotherapy (SC) has the potential to be curative for patients with CLM, as this therapeutic approach has been associated with 5- and 10-year survival rates up to 50 and 35%, respectively (2). Yet, in the recent years, SC has increasingly been used as part of a multidisciplinary strategy to select the most eligible candidates for surgical resection of CLM (3–5). Innovative and effective SC regimens, including oxaliplatin-based and irinotecan-based therapies with or without biological therapies using anti-vascular endothelial growth factor (VEGF) or anti-epidermal growth factor receptor (EGFR) inhibitors have changed the natural history of CLM (6). This has led to the opportunity to offer hepatic resection to a significant proportion of patients with advanced tumor burden, who would not have been considered for surgery until few years ago. To date, different score systems showing heterogeneous results have been developed to stratify prognosis of CLM patient’s undergone hepatic resection (7–9). Nevertheless, in the era of precision medicine, there is still a strong need of new and reliable prognostic markers to cope with the high heterogeneity of CLM that is responsible for a wide spectrum of clinical presentations and different degrees of responsiveness to therapies. Among the variables contributing to the different clinical outcomes of CLM, host immune responses certainly play a pivotal role (10–13). In this article, we review the prognostic factors for patients undergoing hepatic resection for CLM focusing on the emerging shifting paradigm that concerns the role of the host immune system.

## Demographic Prognostic Factors

Gender and age are usually tested as prognostic factors in clinical studies. In general, gender does not significantly impact the outcome (2, 14, 15), while age may have more influence. Yet, advanced age may be associated with increased operative risk and consequently with postoperative complications. Patients with complicated postoperative courses as well as with decompensated comorbidities are unlike to be able to complete the therapeutic program such as to start postoperative SC (16–19). Notably, a recent study on a large multicenter cohort of resected patients showed that age does not affect the long-term prognosis even if after 6 years from the hepatic resection the probability to be cure from this disease starts to increase for younger patients while starts to decrease for older patients. This was due to the onset of other age-specific causes of deaths (2).

## Prognostic Factors Related to the Primary Colorectal Tumor

Many different factors related to the primary colorectal tumor are known to impact the long-term prognosis of resected CLM patients. Table 1 details such factors. Apart from T-status, N-status, and poor differentiation, which are two well-known prognostic factors (14, 17–20), it is interesting to note that recently there was a countertendency about the location of the primary tumor. Historically, tumors in the rectum were thought to be the most aggressive (7, 8, 14), but recently some studies underlined how tumors in the right colon may be the most aggressive (21). Indeed, it has been shown that the embryonic origin of the colon cancer (midgut versus hindgut) may be associated with different pattern of response to preoperative therapies and consequentially to different rates of survival. However, a note of caution should be considered when comparing such different studies, in which the proportion of synchronous CLM is different and the amount of missing data—specifically about the primary colorectal tumor data—is unknown. Yet, a true comparison would require at least a retrospective case–control study with a very large cohort of CLM patients with the same primary and secondary tumor burden submitted to the most similar systemic and loco-regional treatments.

**Table 1:** Traditional prognostic factors of colorectal liver metastases patients.

Factor	Prognosis
Age (older versus younger)	+/−
Sex (male versus female)	=
Embryonic origin (midgut versus hindgut)	−
Advanced T-stage	−
Positive N-stage	−
Poor grading	−
Elevated carcinoembryonic antigen	−
Presence of extrahepatic disease	−
Elevated number of liver metastases	−
Size of liver metastases (larger versus smaller)	−
Synchronous versus metachronous disease	−

## Carcinoembryonic Antigen

Even if disparate results are reported in the literature about its role, the level of carcinoembryonic antigen (CEA) is traditionally considered of importance both for the diagnosis as well as for the prognosis of CLM patients (7, 8, 22–24). Indeed, CEA level was found to be an independent factor correlated with 5-year overall and disease-free survival (22, 23, 25). As a matter of fact, CEA is routinely used in the daily clinical practice to monitor the presence of disease recurrence and to assess the response to SC (23, 25). CEA represents the serum marker used as a surrogate of the biology of the disease. And, in the lack of more personalized and more sophisticated markers, it should be always tested.

## Prognostic Factors Related to the CLM

Table 1 details the traditional prognostic factors of CLM patients. Tumor number, tumor size, bilobar involvement, and status of the resection margin affect the survival (2, 14, 15, 26). Even if it is reasonable that these variables affect survival, they are of limited utility in the daily clinically practice. The use of only these variables does not allow to stratify the long-term outcome especially in the current era of modern and efficient SC (15, 27, 28). Similar considerations may be done for the histopathologic features, such as fibrous pseudocapsule formation, the degree of fibrosis around the metastases, single- versus confluent-node growing, infiltrative versus expansive type of growth, and invasion of intrahepatic vascular structures (29–31). All these features are important and informative, but they do not have an immediate translation in the daily clinical practice.

In general, metachronous CLM are associated with better outcome in comparison with synchronous CLM (14, 15, 32–34). However, many other studies reported similar outcome for synchronous and metachronous CLM (26, 27, 35, 36). This contradictory result may be explained with different types of therapies, either systemic or loco-regional, and different adopted definitions of metachronous CLM. Notably, in another large multicenter cohort of resected CLM, synchronous CLM resulted to significantly and independently impact the overall and disease-free survival (2). However, synchronous presentation of CLMs should not affect the decision to operate, but should influence the timing of resection, particularly in patients at high risk for recurrence meaning that perioperative SC should be always considered in those patients.

## Surgical Margin

It is noteworthy to affirm that the determination of margin width should be based on intraoperative ultrasound findings rather than on the liver palpation or just on what can be seen on preoperative images (37–39). Having said that, the surgical margin of CLM is always a subject of debate among experts. If microscopic negative margin (R0) is desirable in liver surgery, it should be considered the primary endpoint in any liver resection, and in the past years some works have supported that microscopic positive margin (R1), either parenchymal or vascular, may be oncologically adequate in some specific subgroups of patients (38, 40, 41). Conversely, other authors have showed that R1 resections should be avoided being associated with decreased survival (7, 25, 42–46). In a large cohort of CLM patients, Pawlik et al. (47) reported that 1 mm of negative margin was enough to ensure a good outcome. Nowadays, experts agree that the biology of the disease rather than the millimeter of the surgical margin dictates the prognosis of CLM patients (48). In regard to R1 resections, in particular, with regards to R1-vascular resections that are the detachment of the tumor from major intrahepatic vessels, it should be noted that they were found to be oncologically adequate as R0 resections (41). Moreover, those types of resections were conducted on patients otherwise marginally resectable because of advanced intrahepatic tumor burden. In other words, R1 resections may be better than no resections (41). Finally, it should be noted that the comparison among different CLM patients belonging to different studies is difficult. The differences in SC regimens, in intra- and extra-hepatic tumor burden, and in the statistical prognostic models used may, at least in part, explain the different conclusions (49, 50).

## Extrahepatic Disease

The presence of extrahepatic disease, especially if multiple, represents not only a bad prognostic factor but also a contraindication for liver resection. As for the surgical margin, also for extrahepatic disease the literature includes studies with disparate results. Elias et al. (51) showed that the number of CLM may be prognostically more important than the site of extrahepatic disease. Conversely, other authors reported that the site of extrahepatic disease should be taken into account (52). Yet, it is generally accepted that the presence of lung metastases is better than distant lymph nodes metastases (53). A given patient with lung metastases, even multiple, may be treated with wedge resections and achieve adequate survival (54–57).

## RAS and RAF Genes

KRAS, HRAS, and NRAS, belong to the family of GTPases. When activated, KRAS can induce a cascade of mitogen-activated protein kinases that transfers signals from the cell membrane to the nucleus. The RAS gene products activate proteins in the RAF family, which consists of the ARAF, BRAF, and RAF-1 members (58). The importance around RAS mutations, rely on the possibility to predict resistance to the EGFR-targeted monoclonal antibodies. Such resistance may be seen in early stages as well as in advanced stages of the disease with an estimated prevalence up to 44% of CLM patients (59, 60). Several authors have reported significant associations between RAS mutations and survival showing that its molecular determination should be nowadays part of the CLM patient evaluation (61–65). In regard to BRAF mutations, they are reported as low as up to 2% of CLM patients (58). Nevertheless, has been shown that such mutations are independently associated with worse survival (58, 66–68).

## The Role of the Host Immune System

Among the variables contributing to different clinical outcomes of tumors, the host immune responses certainly play a key role (10, 11). Under homeostatic conditions human adult liver contains 10^10^ lymphocytes, with the majority of these cells being cytotoxic T and NK cells (69, 70). Healthy human liver is a dynamic organ, undergoing constant inflammation in the maintenance of homeostasis (71). Elevated levels of chemokines, such as CCL5, CCL2, and IL-8 facilitate leukocyte accumulation (72) and correlate to the increased levels of innate T cells, NK cells, and monocytes seen in healthy liver (73). Yet, others and we have reported that a highly functional and unique subset of NK cells physiologically resides in the hepatic sinusoids and these cells can comprise up to 50% of the total hepatic lymphocyte population (70, 74). Hence, understanding the mechanisms inducing the infiltration of these anti-tumor cells into the CLM is highly relevant both for the estimation of the prognosis and for the development of novel and more effective therapies. A large amount of data that are available in the literature support that the infiltrating leukocytes have a major effect on the clinical outcome (13, 75–79). The CLM patient is not an exception. The tissue microenvironment of metastatic liver is characterized by increased levels of inflammatory cytokines and perturbation of chemokine expression. Molecular cross-talk between tumor-infiltrating immune cells, local tissue-resident immune cell populations, stromal cells, and malignant epithelial cells determines the success of metastatic disease (80). This increased local inflammation may disrupt the normal hepatic immune cell repertoire and subsequent tumor surveillance (81). And, the presence of tumor-infiltrating immune cells has been associated with prolonged survival in patients with colon cancer (12, 13, 82–86). Consistently, it is in fact well known that human liver is highly enriched of cytotoxic anti-tumor lymphocytes including T and NK cells that exert immune-surveillance against cancer (70, 87, 88). Recently, also our group has showed how the presence of intratumoral infiltration of NK cells was an independent prognostic factor favoring overall survival after resection of CLM (89). Those data and results support how the host immune system represents an emerging hallmark of cancer (90) which should not be any more left to exploratory translational researches while it should be routinely considered in daily clinical practice. Indeed, in the past years the tumor microenvironment has been target of many studies aiming to find new promising therapies for solid tumors. Immune checkpoint inhibitors, such as ipilimumab and nivolumab have been introduced also for CLM patients with interesting findings (91). Yet, understanding the biological mechanisms by which a given host immune contexture might be more favorable against colorectal cancer and CLM is the fundamental step that must be achieved to introduce innovative immunotherapies. More efforts should be done to identify which immunological factors are related to the tumors cells, which are related to the host, and how those factors interact to each other. Last but the not least, the role of loco-regional and SC in altering such factors should be one of the researchers’ priorities. Yet, in large cohort of colorectal tumors, a gene–gene correlation network was built to understand which genes may be associated with anti-tumor response (92). As many as 65 different genes were identified, but interestingly most of them were linked to the host immune functionality (T and B cell activation, inflammatory response, T and B cells differentiation, adhesion- and migration-associated chemokines, activation of NK cells), which mean that certainly the immune system deserves further attention.

Table 2 details the immune intrigue that may be part of the liver tissue of CLM patients. Each of the cells represents a potential target for decoding the clinical heterogeneity of CLM patients, for developing new biomarkers, and consequently for developing new personalized immunotherapies.

**Table 2:** Immune intrigue of colorectal liver metastases patients.

Cell type	Location	Role	Prognosis
T cell (CD4+ and CD8+)	Intratumoral; invasive margin	Specific tumor cell killing activity Release cytotoxic cytokines	+

Treg	Intratumoral; invasive margin	Suppress anticancer immune responses	−

Macrophage	Intratumoral; invasive margin	M1: tumor suppression and immune stimulation M2: malignant progression and suppressed CTL	+/−

NK	Intratumoral; invasive margin	Cytotoxic activity and cytokine production	+

MDSC	Intratumoral; invasive margin	Repression of the effector function of T lymphocytes and NK cells	−

DC	Intratumoral; invasive margin	Processing and presentation of tumor-associated antigens	+

*Treg, T regulatory; CTL, cytotoxic T lymphocytes; NK, natural killer; MDSC, myeloid-derived suppressor cell; DC; dendritic cells*.

Notably, myeloid-derived suppressor cells (MDSCs) are immature myeloid cells present in tumor-bearing hosts and can be subdivided into monocytic MDSCs (M-MDSCs) and granulocytic MDSCs (G-MDSCs) (93). Within tumors, M-MDSCs, but not G-MDSCs, rapidly differentiate into TAMs (94). Therefore, TAMs may originate from monocytes, by local proliferation, and from M-MDSCs.

Nielsen et al. (95) postulated a potential role in colon cancer also other immune cells, such as mast cells. Indeed, those cells were found to be independently associated with favorable outcome in a large series of patients. However, data are limited and further studies on mast cells should be carried out.

Interestingly, there are also associations between angiogenesis and the immune system. Notably, the significance of angiogenesis as a prognostic factor has already been investigated in colorectal cancer (96). VEGF plays a key role in angiogenesis, a highly complex process that is essential for tumor growth. Studies showed that VEGF has a significant prognostic role by affecting the tumor’s metastatic potential and by correlating with response to treatment and survival (97). Two signaling pathways play important role in the growth and metastatic potential of human colorectal cancers including the VEGF and EGFR pathways. EGF is one of the natural ligands of the EGFR, which is a transmembrane tyrosine kinase receptor critical to normal cell proliferation and differentiation. An increased level of EGFR seems to be an important factor driving the aggressive behavior of cancer cells (98). Several studies showed a relationship between high EGFR levels and high-grade tumors and poor prognosis (99). And in line with this, some experimental data showed that the blocking of the EGFR pathway was associated with an increased immune infiltration in solid cancers (100, 101). In this regard, several *in vitro* and *in vivo* models have shown that the masking of EGFR with a specific blocking monoclonal Ab (mAb) inhibits tumor proliferation, induces terminal cellular differentiation, and modulates chemo- and radio-sensitivity (102). Recently, it has been also reported that the inhibition of the EGFR signaling pathway facilitates the activation of immune cells and their recruitment to tumor sites *via* the production of several cytokines and chemokines (100). This is particularly relevant for NK cells, as studies have shown that the use of a blocking anti-EGFR mAb stimulates these innate immune effector lymphocytes and induces antibody-dependent cell cytotoxicity. At the same time, the refractory effect of the tumor against this biological compound may be explained by the induction of mechanisms that the tumor can use to evade immune responses. Pre-clinical *in vivo* models have shown the existence of inducible mice carrying altered oncogenic and immunological pathways that are resistant to the inhibition of EGFR (103–105). Hence, since neo-adjuvant therapies in CLM adopt conventional chemotherapy agents (i.e., oxaliplatin and irinotecan), which are associated with biological drugs targeting different pathogenic signaling pathways in CLM, clinicians have to consider all possible mechanisms of oncogenic and immunological tumor escape in order to provide the most effective and customizable therapeutic options.

## Conclusion

Survival following resection of CLM depends on several clinical, pathological, and molecular factors. Apart from those factors related to the tumor, both the primary colorectal and the CLM, there is the need to accurately consider also those factors related to the host immune system, which certainly plays an important role. Yet, the burgeoning data about the immunological intrigue of CLM patients deserve to consider on a single-patient basis with the aim to be more sensitive in the prognostication as well as to introduce more efficient immune therapies. Thus, the sampling and analysis of the tissue microenvironment of CLM is essential and should be part of the standard histological examination of such patients.

## Author Contributions

MD: conception and design. MD, AL, and LT: methodology. MD, AL, LT, CS, and BF: review of the literature. MD: writing, review, and/or revision of the manuscript. MR and GT: study supervision.

## Conflict of Interest Statement

The authors declare that the research was conducted in the absence of any commercial or financial relationships that could be construed as a potential conflict of interest.
